# Shaping Beam Profiles Using Plastic Optical Fiber Tapers with Application to Ice Sensors

**DOI:** 10.3390/s20092503

**Published:** 2020-04-28

**Authors:** Kostas Amoiropoulos, Georgia Kioselaki, Nikolaos Kourkoumelis, Aris Ikiades

**Affiliations:** 1 Physics Department, University of Ioannina, 45110 Ioannina, Greece; kamoirop@cc.uoi.gr (K.A.); georgiakioselaki8@gmail.com (G.K.); 2 Department of Medical Physics, School of Health Sciences, University of Ioannina, 45110 Ioannina, Greece; nkourkou@uoi.gr

**Keywords:** plastic optical fiber tapers, beam profile, ice sensor

## Abstract

Using either bulk or fiber optics the profile of laser beams can be altered from Gaussian to top-hat or hollow beams allowing enhanced performance in applications like laser cooling, optical trapping, and fiber sensing. Here, we report a method based on multimode Plastic Optical Fibers (POF) long-tapers, to tweak the beam profile from near Gaussian to a hollow beam, by generating surface irregularities on the conical sections of the taper with a heat-and-pull technique. Furthermore, a cutback technique applied on long tapers expanded the output beam profile by more than twice the numerical aperture (NA) of the fiber. The enhanced sensitivity and detection efficiency of the extended profile was tested on a fiber optical ice sensor related to aviation safety.

## 1. Introduction 

Shaping beam profiles from Gaussian to top-hat or hollow-beams has attracted a lot of attention due to the numerous applications in lithography, laser processing, fiber optic coupling, optical trapping, and medical research [1,2]. Beam profiles can be altered in a number of ways: using fiber optics with end-facet shaping [3], coupling light in misaligned fibers [4], using interference in multimode fibers (MMF) [5], spiral rays in MMF [6], and by wedge prisms [7]. Also, a top hat-beam beam was generated in a short single mode fiber (SMF) taper, by exiting cladding modes in the short section after a taper and wavelength tuning [8]. Hollow beam profiles have also been generated in MMF using paraxial modes, generated by off-axis coupling and angle coupling in short fiber sections [9,10]. Moreover, beam expansion has been applied to fiber optic solar lighting applications, to illuminate internally large areas in building [11]. However, a particularly interesting application which benefits from tailored beam profiling is optical ice sensing in aviation, due to the requirement for scanning wide angles in scattering media [12]. Adiabatic biconical SMF tapers, or microstructure fibers, have been studied extensively for telecom applications such as coupling light in and out of micro-rings or micro-spheres devices, whispering-gallery modes, frequency filtering and fiber sensing [13,14,15]. They usually have an initial outer diameter (OD) of 125 μm, which is reduced to 1 μm or less and consist of a long thin section in-between adiabatic down and up conical regions with small cone-angles. This geometry permits the fundamental guided mode in the taper to remain stable, without transitioning to higher order modes. Furthermore, the core to cladding ratio is about 1/20, which remains near constant in the bi-conical tapered section. Conversely, POF MMF have a core to cladding ratio of about 24 (240/10) and are mainly used as efficient sensors [16,17]. 

We report the development and characterization of a multimode POF fiber taper, fabricated in a large diameter fiber with thin cladding using a heat-and-pull technique to form long bi-conical tapers, which are cut near the waste to produce two single tapers. Specifically, the adopted heating and pulling protocols alters the surface quality of the conical section transforming the Gaussian modal intensity to a hollow-beam with a much wider beam profile. In contrast with previous work reported in the literature [9,10], which relied on the excitation of paraxial modes in short MM fibers, our method involves coupling a Gaussian beam on the axis of the POF fiber, transforming it at the output conical section of the fiber taper, without any specific fiber length requirement. Additionally, by shortening the taper it was possible to alter the hollow beams to a wide, near Gaussian, profile with an effective output angle two-and-half to three times that of the normal flat polished fiber. This is particularly useful in fiber based solar illumination applications [11] as well in optical ice detection sensors on aircraft wings which benefit from the large numerical aperture (NA) [12].

## 2. Materials and Methods

### Experimental

**Figure 1:**
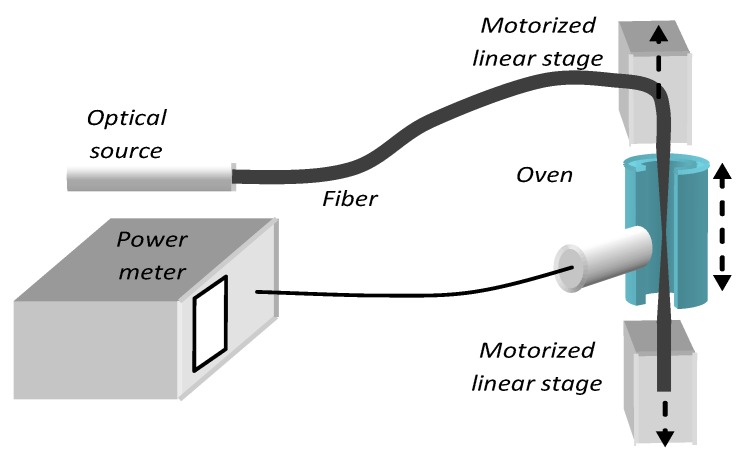
Fiber taper drawing setup.

We used PMMA-based POF fibers purchased from different manufacturers having OD of 250 μm and very thin cladding of 10 to 12 μm (core diameter of about 240 μm). Their refractive indices ranged from 1.45 to 1.47 with a NA of 0.35 to 0.47, transmitting in the visible at peak wavelengths ranging from 400 to 650 nm. The tapers were made by heating and pulling a small section of fiber, in a cylindrical temperature-stabilized electrical oven, to its transition glass temperature (T_g_) near 25 °C to 135 °C. The heater was made of brass having dimension off 25 mm long and a diameter of 2.5 mm with a slot of 0.5 mm to allow free access of the fiber. The heating and elongation assembly were mounted vertically on a motorized linear translation stage, which executed linear or oscillatory motion to increase the heated volume. Two vertically mounted, computer controlled, linear stages pulled the fiber as shown schematically in Figure 1. The pulling stages could move in opposite directions synchronously or not at different rates. The setup permitted different geometric profiles of bi-conical tapers to be obtained by adjusting the pull rate of the stages, the temperature and the position of the heater. 

**Figure 2:**
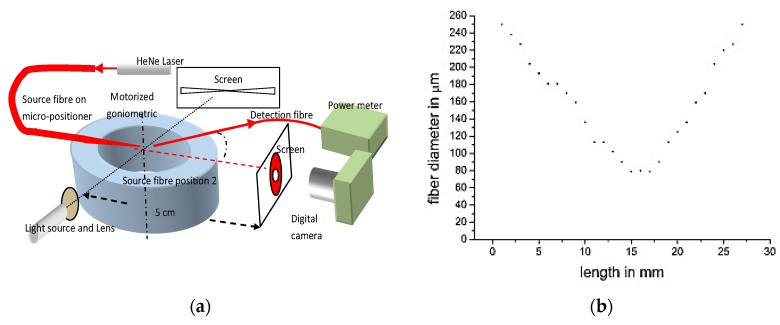
(**a**) Experimental arrangement used to measure the taper geometry, (**b**) Fiber taper measurements side-profile.

With some elongation protocols the bi-conical tapers became progressively lossy, radiating light from the conical down-taper and the thin section of the fiber taper. The optical losses increased as the tapers became thinner and longer. Thus, we monitored the fabrication process, by measuring the light exiting from the side of the biconcave taper as shown in Figure 1. The loss mechanism was subsequently investigated and will be discussed in the following section. The measurement of the taper geometry, and beam profile, was combined in one setup, shown schematically in Figure 2a, using an optical projection method, which magnified the fiber side-image on a screen. The images were photographed with a digital camera and analyzing using Image-J (https://imagej.nih.gov/ij/, v.1.52, U.S. National Institutes of Health, Bethesda, MD, USA). A typical taper side-profile measurement is shown in Figure 2b. 

**Figure 3:**
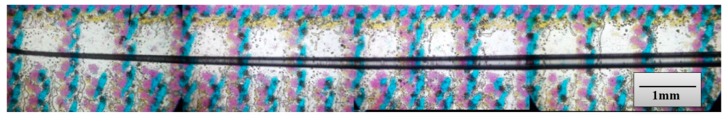
Microscope picture of 10 mm long POF tapper with initial OD of 250 μm.

Bi-conical tapers were subsequently cleaved at the taper waste as shown in Figure 2b, producing two tapers with approximately 1.5 m long fiber necessary for excluding input cladding and paraxial modes (Figure 3). 

The fiber tapers were cleaved at the tip and clamped in the center of the goniometer as shown in Figure 2a. A laser beam (530 nm) was coupled, on axes, at the input end of the fiber and exited from the taper. The output beam profile was mapped using a motorized goniometric-setup consisting of a 400 μm OD detection-fiber, with polymer cladding and glass core, NA of 0.45, which was positioned on the rotating arm. A power meter measured the coupled intensity at the output end of this detection fiber (Figure 2a). The center of rotation of the detection fiber, was set at about 6 mm from the tip, and rotated 180° along the horizontal plane, measuring the angular intensity of the beam. Furthermore, the beam exiting the taper was projected on a screen and photographed using a digital camera, set at about 5 cm away, for beam symmetry diagnostic and documentation purposes. 

## 3. Results and Discussion

### 3.1. Optical Losses from Tapers and Beam Profiles 

**Figure 4:**
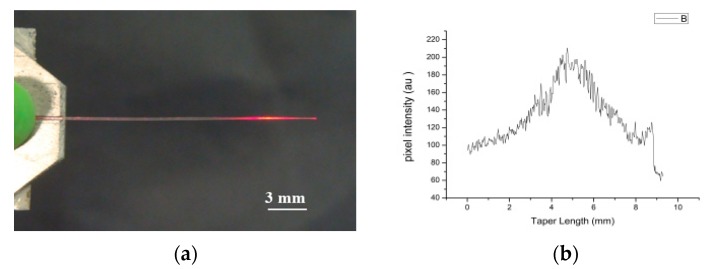
(**a**) Side view (perpendicularly to the taper axis) of illuminated taper, (**b**) Side intensity distribution.

Several heating and elongation tests were carried out with pulling rate varying from 0.5 to 13 mm/min, while keeping the oven either stationary or moving at a rate of 5 to 15 mm/min over a distance of 20 mm. The pulling rates were determined by two parameters: (i) the initial tendency of the fiber to contract, due to memory processes in the PMMA which set the lower pulling limit and (ii) braking of the fiber when the pull rate exceeded the rate at which the fiber-core dropped below T_g_. The taper lengths ranged from 6 mm to 17 mm and formation of asymmetrical conical sections were observed when only one stage was allowed to move. As mentioned above, tapers drawn near T_g_ and at about 350 to 400 μm/min at temperatures of 125 to 135 °C, became lossy, radiating from the conical section (Figure 4a). The intensity variations along the taper length are depicted in Figure 4b, with most of the light exiting midway of the conical section indicating that the modes were no longer guided in the taper but radiated in nearly 2π steradians. 

**Figure 5:**
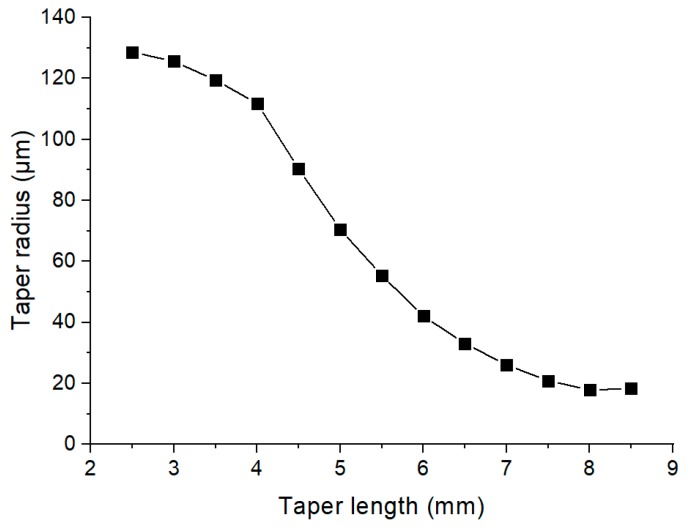
Typical taper profiles with radii 20 to 45 μm.

The physical dimensions of the tapers were measured from the shadowgraphs using Image-J. The length of the conical sections was defined as the points were the diameters at the thin and thick sections were respectively, 10% and 90% of the final values, and ranged from 6 to 17 mm. The corresponding conical angles were from 1.0 ° to 2.5° and waists diameters from 30 to 90 μm with a typical profile shown in Figure 5.

**Figure 6:**
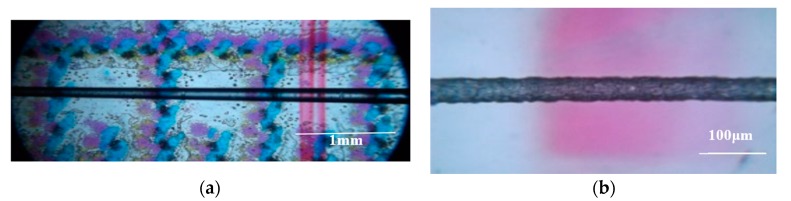
(**a**) Microscope pictures of fiber down-tapers section magnified x10, (**b**) along the taper waist magnified ×40.

Under an optical microscope, the bi-conical fiber tapers exposed surface imperfections (i.e., anomalous surface, AS). In Figure 6a,b, a 3 mm conical section and a section near the tip of the taper with diameter of about 35 μm, are shown respectively. The length of AS did not cover the entire taper (Figure 6a) but progresses in the thinner sections covering a few mm in the down-taper, extending into the taper waist, covering 40% to 50% of the taper length. By correlating the lengths of AS on the conical surfaces of the tapers with the intensity profile measured with Image-J (Figure 6b), it was apparent that optical loss coincided with the AS region. This finding verified that the output modes were no longer guided by Total Internal Reflection (TIR) but undergone diffuse scattering by AS. Furthermore, light was diffused out of the taper well before the end the AS region. 

**Figure 7:**
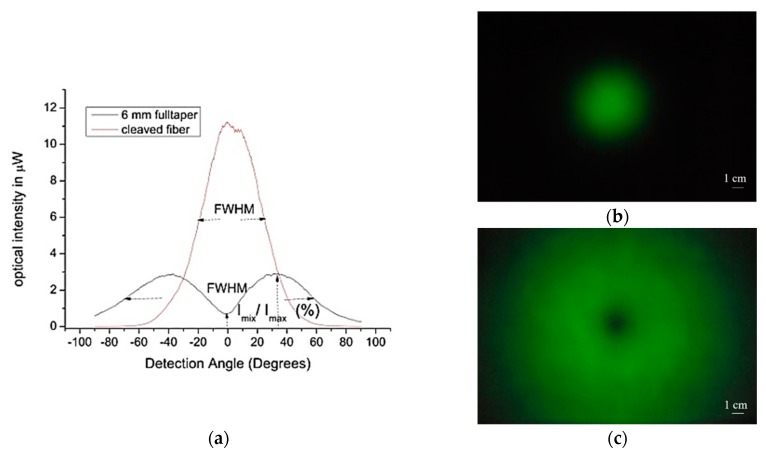
(**a**) Goniometric measurements of beams from a taper (twin peaks) and a flat polished fiber (single peak (**b**) Picture of beam profile from a flat polished fiber, projected on a screen, (**c**) and that of a taper.

The output modal intensity profile from the tapers were measured using the goniometric set up, as described earlier in Figure 2, together with the beam profile from a flat polished fiber, used for comparison. Typical goniometric beam profiles for a polished fiber and a taper are shown in Figure 7a. The modal intensity profile from a polished fiber and a typical 15 mm long taper were projected on a 25 cm by 17 cm semi-transparent screen and photographed with a digital camera (Figure 7b,c).

The “effective” diameter of hollow beams was measured at the full width half maximum (FWHM), with the maximum being defined relative to the average intensities of the twin peaks, as indicated in Figure 7a. The goniometric measurements for normal 250 μm diameter polished fibers with NA of 0.38 were compared with measurements of a typical 6 mm long fiber taper with cone angle of about 1.2°, yielded a FWHM values of 45° and 130°, respectively. Similarly, the central intensity-depression (ID), or dark spot, was measured as the percentage ratio of the intensity at the center, relative to the average intensities of the twin peaks (Figure 7a). 

### 3.2. Background on Beam Profiles from MMF Tapers 

**Figure 8:**
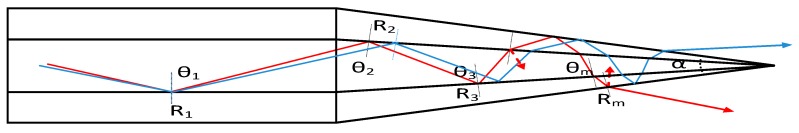
Ray optics in smooth MM fiber taper.

To better understand the origin of the side losses it is necessary to model the expected exit angles from a long smooth MMF taper using the meridian ray optics model. In this first approximation model, the paraxial modes are not included (a) due to the large diameter of the fibers and (b) because the Gaussian modal intensity profile implies the light energy is concentrated in the center of the beam (Figure 7b). Therefore, ray-optics theory applied on meridian rays can adequately describe the light propagating in the large core fiber tapers and predict the formation of hollow beams with low exit angles in the conical section of the taper [16]. When calculating ray optic paths in the fiber, cladding modes can be neglected due to very thin cladding in the conical section of the taper. In Figure 8, meridian rays enter a taper with cone angle *α,* undergoing successive total internal reflections (TIR), which gradually reduce the reflected angle at the interface. Eventually the rays will exceed the critical angle and will be converted to cladding modes refracting out of the fiber taper as radiation modes [16,17,18]. If we consider a meridian ray guided in the conical section, the reflected angles at the interfaces, R_1_, R_2_, ..., R_m_, change as shown schematically in Figure 8. At the interface, the rays’ angle can be calculated for an initial angle θ_1_ as:

(1) $$\theta_{m+1}=\theta_{1}-\alpha \left(2m-1\right)$$

where m = 1, 2, 3 …, are the number of successive reflections at the taper boundaries.

**Figure 9:**
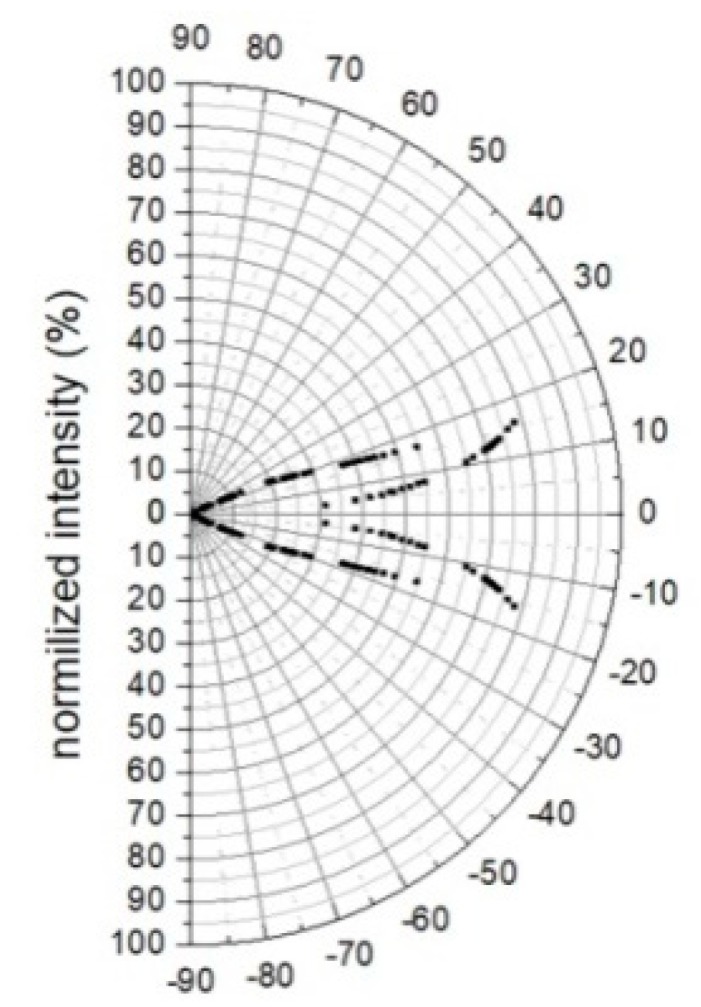
Ray optic modeling of angular intensity distribution of radiation modes exiting from the sides of the fiber taper (α = 2°).

Consequently, the optical transmission intensity can be modeled for a long taper. Typical results in polar coordinates are shown in Figure 9 with cone angle *α* ranging from 1° to 3°. This theoretical model ignores the thin cladding which eventually becomes even thinner by the drawing process shortening the optical paths. Therefore, the model predicts that when shaping a hollow beam, most of the light escapes from the sides of the smooth taper in angles 5° to 17° to the fiber axis while no light exits from the tip of the fiber (Figure 9). 

However, the calculated profile of beam is much narrower than the experimental, as depicted in Figure 7, with exit angles of the two peaks at about ±45° to ±50°, corresponding to a much wider hollow beam profile. This discrepancy is attributed to the AS, which changes the beam profile due to diffuse scattering at the surface of the taper and cannot be included in the theoretical model. Although it is beyond the scope of this work, the formation of the AS can be attributed to the differences in dopants levels required for the refractive indices of the core and cladding, that result in varied T_g_ [18]. An alternative explanation could be deferential heating due the heater geometry and the size of the slot. During drawing at T_g_ = 130 °C, the two layers undergo different relative motion leading to the AS which increased optical losses from this region of the taper. 

**Figure 10:**
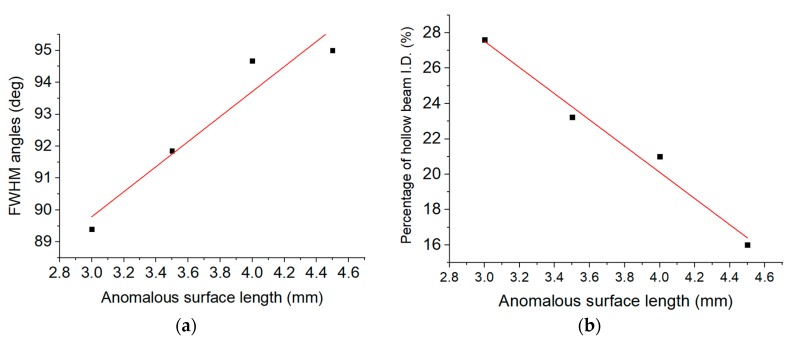
(**a**) Angular FWHM is plotted as a function of AS length in mm, (**b**) Central intensity-depression (dip) of hollow beam as a percentage of the maximum values and the AS length in mm. Each point in the two graphs corresponds to four tapers with similar AS lengths.

Experimentally, two parameters are used to characterize the hollow beam profile, namely the FWHM of the tween peaks and the ID of the beam. A positive correlation of the A.S length with the beam profile is shown in Figure 10a. Furthermore, the depth of ID was negatively correlated to AS length as shown in Figure 10b. Based on these results the ID % values of the hollow beam, decrease with the length of the AS. For example, 3 mm length of AS corresponds to ID of 28% of average peaks values while reducing to about 16% when the AS length increases to 4.5 mm. Therefore, as the length of the AS increases up to 4.5 mm, more light exits from the side of the taper generating of a hollow beam with ID as low as 3%. This was also verified by the low throughput transmission (less than 1%), measured before cutting the biconical tapers into two single tapers.

**Figure 11:**
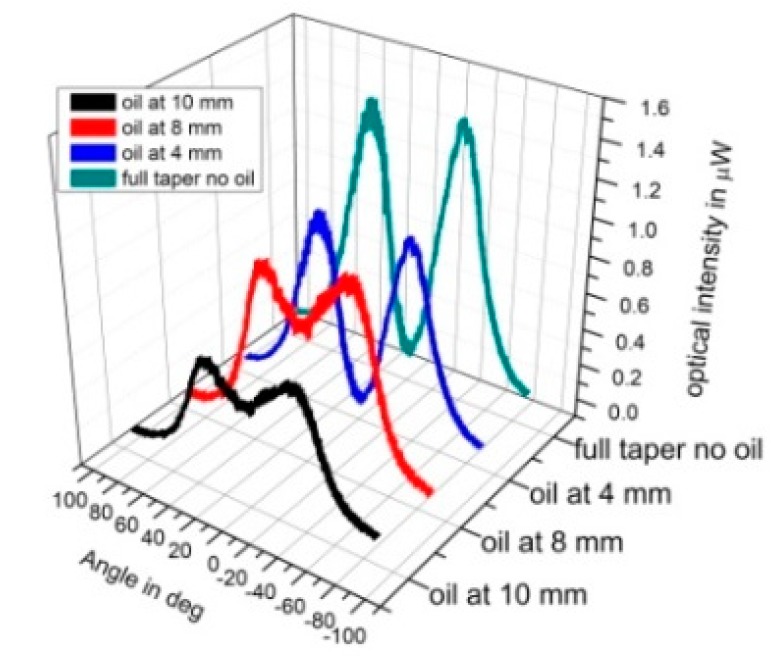
Angular optical intensities for a taper with index matching oil gradually covering the conical section.

To further investigate the loss mechanism in tapers, the AS region was effectively “smoothed” by covering it with index matching oil with refractive index *n* = 1.51. Using a small hyperemic needle, an oil droplet was progressively dragged along the surface of the conical section starting from the tip until all the AS was covered. Figure 11 shows the change to the hollow beam profile, for a 13 mm long taper with about 10 mm of AS, which is progressively covered with oil. As expected, when oil was applied upon AS, scattering is gradually reduced and more light reaches the end of the taper, shaping a beam profile with reduced ID of the central dark spot. 

Specifically, for the full taper the ID is about 3% of the average twin-peak values, gradually increasing to about 75 % when the oil covers the entire AS region. Similarly, the FWHM of the beam is also gradually reduced, due to less scattering from the conical section, reaching lower exit angles of 10° to 17°, when the AS is completely covered, which are close to the predicted values of a smooth taper as predicted by the theoretical model (Figure 9).

### 3.3. Beam Expansion with Cutback 

**Figure 12:**
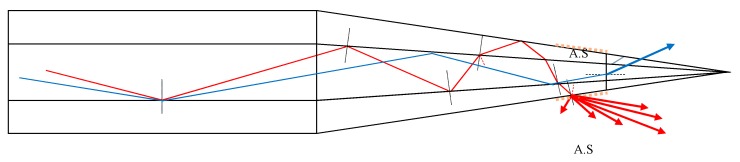
Schematic ray optic diagram of fiber taper cutback and optical diffusion from the side of the AS.

Based on the above results it is evident that the AS on the surface of the taper, causes diffuse optical transmission of the guided modes to exit the taper, changing the optical profile of the beam. In order to quantify this effect further and to investigate the ability to alter the hollow beam profile, the length of the AS region of the taper was gradually shortened. This involved cutting progressively small sections of the taper (cutback), thus altering the ratio of light exiting the side and the cleaved end-facet of the taper, as shown in Figure 12 where the AS length was reduced, allowing the more central guided modes to exit the fiber cleaved-end rather than the side of the taper. 

**Figure 13:**
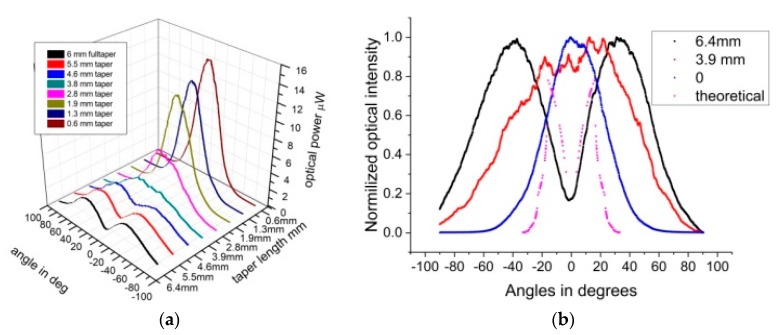
(**a**) Angular intensity distributions for cutbacks full taper 6.4 mm, (**b**) Normalized angular intensity distributions for cutbacks full taper 6.4mm, (black), 3.9 mm (red), normal fiber (blue) and ray optic model (magenta). The *x*-axis shows the angular displacement of the rotating detection fiber in degrees, with the center being at 0°. The *y*-axis represents the lengths of the taper in mm, rather than the length of the AS, which, in this case were similar. The *z*-axis is the optical intensity in μW.

Using the experimental setup outlined in Figure 2a, the taper was placed in the center of the goniometer while the rotating arm was scanning from ±90°. Once the angular scan was completed, a small section of 0.5 mm long was cleaved, and the fiber tip repositioned in the center of rotating setup. The angular measurements repeated until the conical section was completely removed. In Figure 13a, a 3D graph of the angular intensity variations is shown for progressive cutbacks of the taper. For a 6.4 mm long taper, the initial hollow beam profile changed to an expanded Gaussian, and near top-hat when the taper length was reduced to about 3.9 and 2.8 mm, respectively, reverting gradually to Gaussian when all the taped sections together with the AS were removed. 

**Figure 14:**
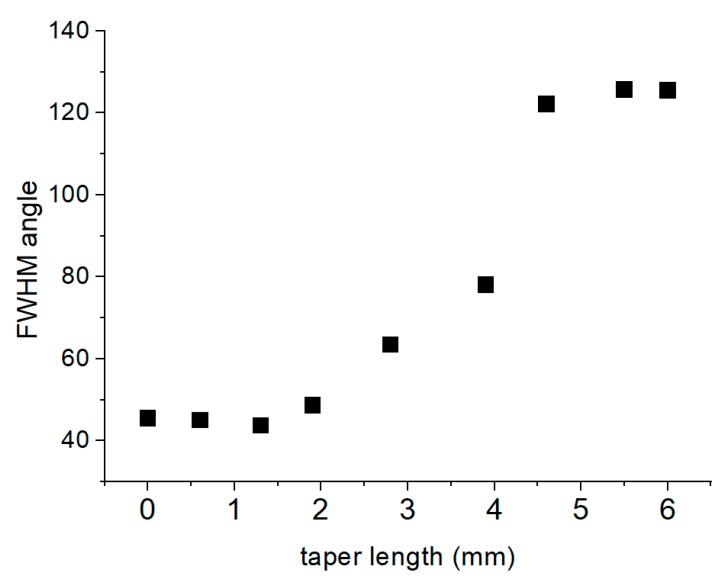
FWHM as a function of taper length with cutback technique. Points represent average values.

The normalized intensity variations as a function of angle for three distinct cutback lengths are depicted in Figure 13b. The black line is for the full taper of 6.4 mm, with the ID having its lowest value of about 17% and effective FWHM of the hollow beam is widest at about 128°. The red line is for the taper shortened to 3.9 mm; the beam is no longer hollow exhibiting a wide profile with FWHM, extending to nearly 100°. The blue trace is the beam profile when the entire taper was cut off with a FWHM of about 45°, corresponding to a fiber with NA of 0.38. The modeled angular distribution for a smooth taper is shown in the doted magenta trace and is similar to that in Figure 9 expressed in linear rather than polar coordinates. The expected exit angle for this taper has FWHM of 32°. Finally, the variation in the FWHM with taper length during cut back, is shown in Figure 14. As a result, with tapers in POF fibers, the beam profile can be tailored as necessary, expanding the normal FWHM of 45° to above 120°. 

### 3.4. Ice Detection with High-NA POF Fiber Tapers

One important application of the discussed beam expansion technique is in the development of a fiber optics ice detector. Ice accumulates on the leading edge of aerodynamic surfaces disrupting the normal airflow and occurs when aircrafts fly through cold moist air. These conditions are particularly dangerous when an aircraft flies at low altitudes near congested airports because lift decreases and drag increases [19,20]. The current technology for ice detection relies on atmospheric conditions measurements like temperature, humidity, and liquid water content (LWC) in order to calculate icing conditions based on algorithms, and alert the aircraft of the ice conditions without directly detecting ice on the wings. Similarly, present icing sensors which are located on the nose of the aircraft, and are based on a vibrating wire which measures the ice mass accreting on the wire, through changes in its resonant frequency, also don’t detect ice directly on the wings [21]. 

**Figure 15:**
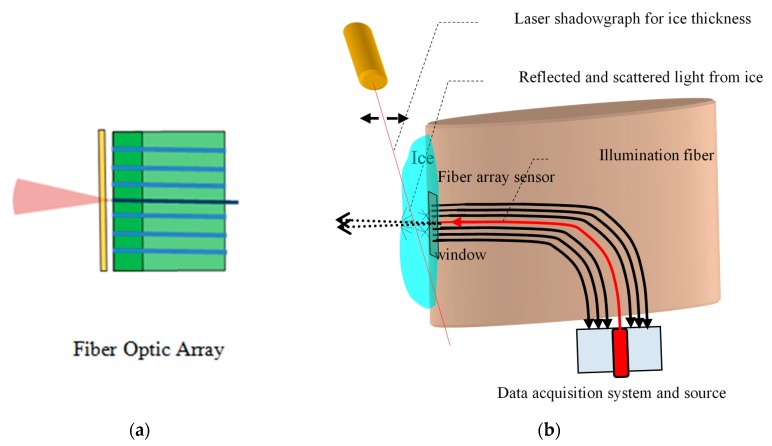
(**a**) Schematic diagram of the ice sensor. (**b**) Experimental lay out in the icing tunnel for calibrating ice thickness.

Previously, we have reported an alternative method for direct ice detection based on optical diffusion which measures, in real time, the thickness and type of ice accreted on the wings of aircraft using a fused-silica fiber optical sensor array [12]. A schematic diagram of the ice sensor and its layout in the icing tunnel is shown in Figure 15a,b.

In principle, the method relies on illuminating and detecting the light from the accreting ice on the leading edge of the airfoil. Depending on the freezing rate of water, gases dissolved in the super-cooled droplets may or may not escape, forming micro-cracks and micro-bubbles in the ice volume. The density of these discontinuities varies, giving ice its optical characteristics, attributed primarily to Mie scattering [22].

The fiber array senor, used in the previous work [12], consists of six multimode flat polished detection signal-fibers, arranged in sets of three, on either side of a central seventh source-fiber with a 2 mm pitch. All the fibers had a NA of 0.1 and the illumination fiber coupled the light from a Laser Diode (LD) emitting at 650 nm (Figure 15a). Light from the illumination fiber is partially reflected and partially backscattered from the surface and ice volume respectively. The diffuse light is coupled to the signal fibers and transmitted to a set of photodiodes, one for each fiber. The fiber array sensor was located on the stagnation line, of a zero-lift wing, placed vertically in an icing tunnel. All tests were conducted with airspeed of about 150 Knots and temperatures ranging from −5 to −25°C with LWC of 0.5 to 2 gm/m^3^. The optical intensity was recorded as a function ice thickness, measured by a shadowgraph technique using a perpendicular laser beam, which traced the front edge on the ice (Figure 15b). 

**Figure 16:**
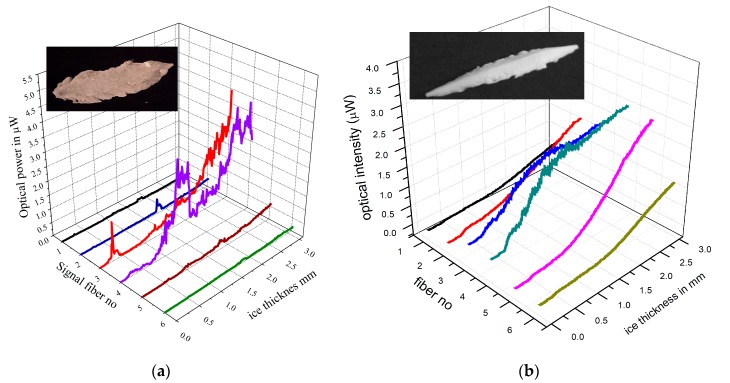
Optical measurements of ice thickness with flat polished fiber array sensor (LWC 0.5 mL/m^3^ and 20 μm water droplets) (**a**) glazed ice (−5 °C) (**b**) rime ice −20 °C.

Depending on the ambient conditions, the accreting ice takes different shapes and forms influenced by the aerodynamics of the airfoil, which generates a local temperature gradient spike over the wing, due to the adiabatic expansion of the air passing over it. A key parameter, which determines the optical characteristics of ice, is the freezing fraction (FF), defined as the ratio of ice that freezes directly on impact to that which remains liquid and freezes behind the impact area [20,22]. The values of the FF range from zero, for water remaining liquid, to one when all water freezes on impact. The local FF determines the type of ice accreted which can either be clear glazed ice for FF close to 0, opaque rime ice for FF close to 1, or mixed phase ice, with varied transparency, and FF with values between 0 and 1. Typical measurement for glazed and rime ice are shown in Figure 16a,b, respectively, were the optical intensity is shown as a function of ice thickness for the six, low NA flat-polished signal fibers. In these graphs, the illumination fiber is in between fiber 3 and 4. The maximum detected ice thickness was restricted to about of 3 mm, which is considered a safe threshold for activating the ice protection systems. 

In glazed ice (−5 to −10 °C), reflected light from the surface of the ice dominates and signals from the central fibers 3, 4 increase rapidly up to an ice thickness of 3 mm, and exhibit acute intensity spikes. The origin of these intensity spikes is primarily due to transient ice micro-crystals, formed on the surface of the thin transparent ice which can be detected by the inner fibers, being closest to the illumination fiber (Figure 16a). Conversely, the signals from the outer fibers 1, 2, 5, 6, rise slower with ice thickness, exhibiting overall lower intensities but are “smoother” as they detect predominantly diffuse reflections and scattered light due to the greater distances from the source fiber (Figure 16a). Similarly, in rime ice (−20 to −25 °C), scattered light dominates, and the intensity distributions are much smoother (Figure 16b) and lack the intensity peaks seen in glazed ice. In rime ice however, backscattering is the dominant mechanism, as reflected light from surface diffuses in the opaque ice, leading to much smoother signals with virtually no spikes (Figure 16b). 

Another interesting point is that for a low NA fibers-array sensor in glazed ice, the backscattered light is near constant for a particular ice thickness, while the strong transient reflections, near the fiber facet will momentarily contribute a much higher percentage of the detectable power, thus distorting detectible signals. Conversely, if the acceptance angel is increased, light from a much wider “field of view” will be detected, so transient events will represent a lower percentage of the detectable signal, thus averaging out their influence on the signals. Similarly, if the accreting ice is illuminated by a wider beam profile, the overall near field intensity is reduced, thus also reducing the transient reflections. Therefore, this effect can be achieved by using high-NA, MM-POF tapers, both for illumination and detection, utilizing the cutback method as described above. 

**Figure 17:**
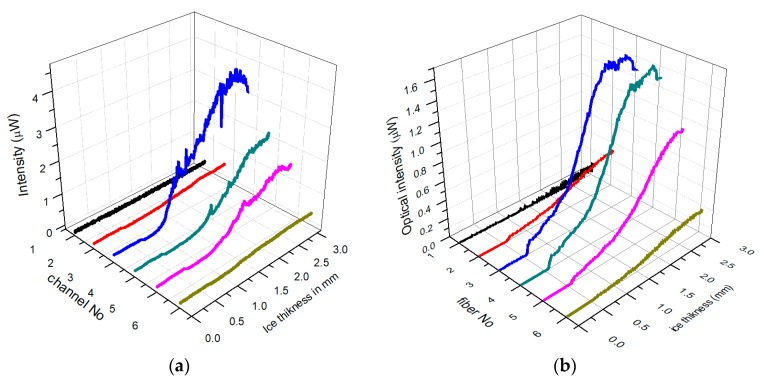
Optical measurements of ice thickness with POF taper fiber-array sensor (LWC 0.5 mL/m^3^ and 20 μm water droplets) (**a**) glazed ice (−5°C) (**b**) rime ice −25°C.

By retaining the same fiber-array sensor architecture shown in Figure 16a and replacing the polished fibers with MM POF cleaved tapers, the detection efficiency of the ice sensor can be considerably improved by enchasing the overall NA to about 0.85, with acceptance angles ranging from 120^°^ to 140^°^. Typical results for large NA array sensor are shown in Figure 17a for glazed ice and Figure 17b for rime ice. 

Similar intensity patens were obtained with the low NA, polished fiber optic array-sensor (Figure 16a,b) and the POF fibers tapers array sensor (Figure 17a,b). However, the intensity distributions of glazed ice are much smoother due to the wider illumination of the ice volume and the effective averaging effect of the wider NA of the detection fibers. The elimination of the intensity spikes improves the signal to noise ratio and therefore, significantly improves the detection of ice buildup rate on the leading edge of the wings. 

## 4. Conclusions 

We have demonstrated the ability a POF taper to tweak the near Gaussian modal intensity profile of a cleaved fiber to a deep hollow beam profile with very low intensity dark spot in the central ID. Our experiments showed that the underlined mechanism of beam shaping was the generation of AS on the side of the taper which disrupted the TIR process on the guided modes, exiting the conical section by diffuse transmission from the side of the taper. Furthermore, by a suitable cutback technique the modal beam profile could be altered from a hollow-beam to near top-hat, and finally to an expanded Gaussian, by cutting back the tapers from 35% to 40% of their original length. This technique redistributes the ratio of the radiation to ending modes, which exit the sides of the taper and the flat polished fiber tip respectively, allowing the beam profile to expand almost 2.5 times the FWHM of the original fiber. Furthermore, we demonstrated the use of the modified POF tapers in ice sensing applications with improved signal-to-noise ratio in all aspects of the detection envelope and in particular in detecting glazed ice.
